# Noseband Use in Equestrian Sports – An International Study

**DOI:** 10.1371/journal.pone.0169060

**Published:** 2017-01-03

**Authors:** Orla Doherty, Vincent Casey, Paul McGreevy, Sean Arkins

**Affiliations:** 1 Department of Life Sciences, University of Limerick, Limerick, Ireland; 2 Department of Physics, University of Limerick, Limerick, Ireland; 3 Faculty of Veterinary Science, University of Sydney, New South Wales, Australia; University of Minnesota, UNITED STATES

## Abstract

Nosebands are used by riders to prevent the horse from opening its mouth, to increase control and, in some cases, to comply with the competition rules. While equestrian texts traditionally recommend that two adult human fingers should be able to fit under a fastened noseband, noseband tightness levels are not, in general, regulated in competition. Possible detrimental consequences for the horse, of excessively tight nosebands, include discomfort, pain or tissue damage. The current study investigated noseband usage in equestrian competition. Data regarding noseband type, position, width and tightness were collected from 750 horses in eventing (n = 354), dressage (n = 334) and performance hunter (n = 62) competitions in Ireland, England and Belgium. Data were collected immediately before or after the performance. Using the ISES taper gauge as a guide, results were classified according to the number of ‘fingers’ that could fit under the noseband at the nasal planum, and assigned to six groups: greater than 2 fingers; 2 fingers; 1.5 fingers; 1 finger; 0.5 fingers; zero fingers. A calliper was used to measure noseband width and position relative to the facial crest. The data were not normally distributed so Kruskall-Wallis and Mann-Whitney tests were used. In all, 44% of horses fell into the zero fingers classification while only 7% were in the two fingers classification. Significant differences emerged between disciplines (p<0.001), with the highest levels of noseband tightness measured among eventers followed by dressage horses with lowest levels among performance hunters. Noseband tightness did not differ significantly with horse age (p>0.05), which ranged from 4 to 19 years. The flash noseband was the most commonly used noseband (n = 326) and was significantly tighter than the cavesson (p < 0.001), drop noseband (p < 0.001) and the Micklem (p < 0.005). Noseband width ranged from 10 to 50 mm. Noseband position varied widely with the distance between the facial crest and upper noseband margin ranging from 0 to 70 mm. The high proportion of very tight nosebands found in this study raises concerns regarding the short and long term behavioural and physiological consequences of such tight nosebands are for the horse. Although these data are currently lacking, the findings are of concern.

## Introduction

By nature, the horse is a so-called flight animal, responding to frightening situations by fleeing. Many equestrian sports routinely demand fast locomotory responses, which are akin to the flight response and, whether caused by a fear reaction or performed in response to a conditioned cue from the rider, are generally accompanied by high levels of arousal. In equestrian sport, fast locomotory responses in a highly aroused horse can be difficult to control. Injury rates in equestrian sports are high in comparison with other sports (Hawson et al., 2010a), so control of the horse is of paramount importance for the safety of horse and rider. Humans control horses through a combination of restraint and training. Training occurs through the use of negative reinforcement, which relies on the removal of aversive pressure immediately after the desired response has been performed [1].

The bridle is the main instrument for controlling horses throughout the world and is used in all equestrian disciplines. Its use dates back approximately 6000 years and relies on application of pressure to sensitive areas of the horse’s head and particularly in the oral cavity. Bridles vary considerably in design. The width, position of straps and method of tightening of nosebands is one aspect in in which bridles show particular variation. Contemporary nosebands vary in width, design and mechanisms of tightening to offer a range of potential pressures. Many bridles used in the Olympic disciplines (eventing, dressage and show-jumping) now incorporate a Swedish cavesson noseband, more commonly known as the crank noseband. This type of cavesson has a leveraged buckle design that allows the noseband to be tightened to a much greater tension with less force than is possible with the French cavesson, more commonly called the plain cavesson [2–3].

Traditional recommendations about bridle fitting suggest that after the noseband has been fastened, two fingers should fit comfortably beneath the noseband. The origin of this guideline is unknown but it has been appearing in equestrian texts since 1956 [4], up until the present [5–9]. However, the variation in the assessors’ finger size and varying opinions on where the fingers should be placed to assess noseband tightness have been cited as shortcomings of this guideline. Consequently, few, if any, governing bodies or organizers of equestrian events have a process in place to assess and regulate noseband tightness. The Federation Equestre Internationale (FEI) Code of Conduct for the Welfare of the Horse states that ‘*Any practices which could cause physical or mental suffering*, *in or out of competition*, *will not be tolerated*” [10]. However, there is a lack of scientific data on the sensitivity of equine tissues to pressure, the levels of pressure required to activate nociceptors in the skin and soft tissues) and cause pain perception or ultimately, the levels of pressure likely to cause tissue damage. These knowledge gaps make it difficult for any governing body to ensure that noseband use that could cause physical or mental suffering is eliminated.

Apart from noseband tightening, the width and positioning of nosebands may influence their impact on the horse [3]. For a given tightness, narrow nosebands will generate higher pressures on supporting tissues than wide ones. The nasal bones are progressively less broad rostrally than caudally, so the position of the noseband on the horse’s head may influence the extent to which sub-noseband pressure is exerted against bony structures rather than soft tissue [11]. The traditional recommendation regarding proper position of the noseband is that 1.5–2.0 fingers should fit between the upper margin of the noseband and the distal margin of the facial crest [8]. This guideline suffers from the same lack of precision as discussed earlier (in reference to tightness) and is not enforced. Moreover the possible consequences for the horse of variation in noseband position and width have not been investigated.

Equestrian texts acknowledge the need to use pressure on the head in training and controlling horses [12, 8]. The commonly targeted locations for pressure application via bridles include the diastema, tongue, lips, chin groove and poll. The authors are unaware of any reference in authoritative equestrian texts or literature to the use of noseband pressure as an adjunct to the acknowledged sites of pressure control. The magnitude and range of both static and dynamic pressures under nosebands and the distribution of such pressures relative to distinct anatomical features are critical to understanding the control function of such pressures as well as their impact on animal well-being. Some preliminary work has been reported that indicates that as nosebands are tightened, there is increased sensitivity to the bit [13] and an increase in eye temperature, suggestive of stress [2].

A growing awareness of horse welfare has been accompanied by concerns regarding some common traditional practices in equitation [14]. The practice of over-tightening nosebands is of concern to equitation scientists and some veterinarians [15–16]. In dressage, riders are penalised if their horses open their mouths, so there may be an incentive for riders to prevent mouth opening. If this practice increases the riders’ control of their horses, an additional incentive arises. Tight nosebands may have an appeal to riders but may mask undesirable oral activity and increase sensitivity to the bit(s) at the expense of horse welfare. It is possible that tight nosebands cause pain and possible tissue damage as the horse fights against the noseband in attempts to seek comfort through various oral activities.

Preliminary research suggests the possibility that vascular perfusion to the muzzle may be compromised by tight nosebands [2]. As a response to growing concern about the practice of over-tightening nosebands, the International Society of Equitation Science (ISES) designed a simple device (the ISES Taper Gauge) with geometric features analogous to one finger and two fingers [17]. The gauge was designed to allow riders and competition organizers to assess and regulate noseband tightness. The society also issued a position statement advising regulatory authorities and riders to check noseband tightness levels to ensure that the horse is not subjected to excessive pressure [17]. However, over the past four years, the uptake of the taper gauge by equestrian governing bodies has been negligible. This may reflect the lack of available data on the prevalence and possible consequences of excessively tight nosebands. The aim of the current study was to provide data on current noseband design, position and tightness in equestrian competition.

## Materials and Methods

Approval from the University of Limerick Animal Ethics Committee was granted to carry out the study described in this paper (Number 2013_6_1_ULAEC). This research involved measurement of current noseband usage and did not subject animals to any procedure which would threaten or reduce their welfare

### Taper gauge

**Fig 1:**
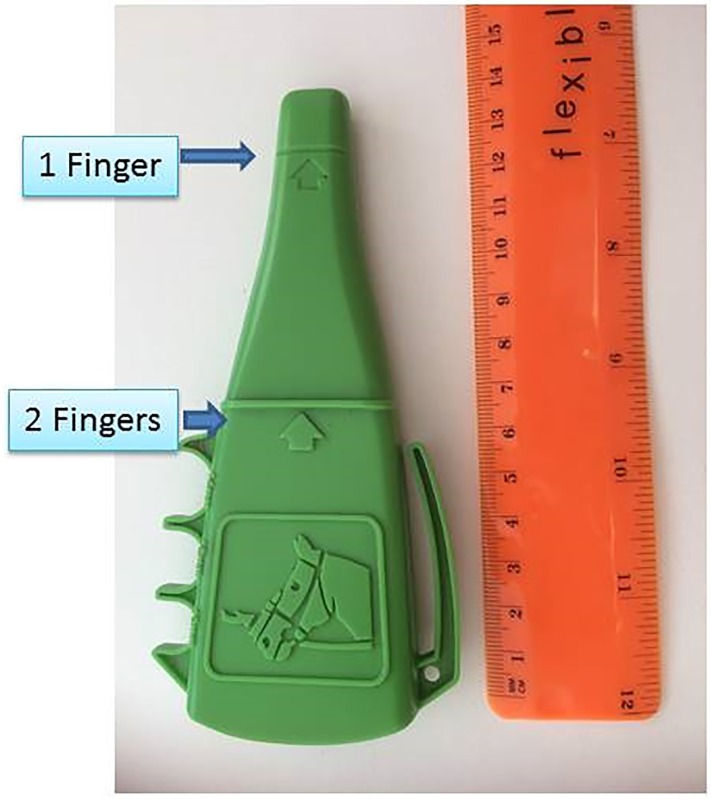
ISES Taper Gauge designed to allow measurement of approximate noseband tightness. At standard recommended noseband tightness, the taper gauge can be easily inserted beneath the noseband as far as the 2 Finger notch.

The Taper Gauge was used to estimate the tightness of the nosebands reported in the current study. The ISES Taper Gauge (Fig 1) was designed to allow riders, coaches and tack inspectors at competitions to measure and regulate noseband tightness levels. It tapers smoothly from one finger diameter to that of two fingers, as determined from a sample of ten adult males and ten adult females, (circumference 102 mm) [2].

### Callipers

Tuberculosis skin thickness callipers (Duggan Veterinary Supplies Ltd, Tipperary, Ireland), were used to measure noseband width and position. These callipers were designed specifically for use on skin and, as such, have blunt, rounded arms that can grasp a fold of skin without causing skin damage and therefore do not pose a risk to the horse in this context.

### Animals

Approval from the University of Limerick Animal Ethics Committee was granted to assess the type and method of usage of nosebands in competition in the disciplines of eventing, show jumping and dressage (Number 2013_6_1_ULAEC). Competitions were selected where the horses were required to undergo a formal tack inspection immediately prior to, or following, their participation in the competition.

### Venues

Data were collected at the following locations:

1. Young Horse Eventing League, Ireland
  Data were collected from horses (n = 139) competing in an annual eventing league for young (4 and 5 year old) horses run one day per week over five weeks at various locations in Ireland. This league is conducted under the regulations of the Future Event Horse League (FEHL). All horses were required to undergo a tack inspection prior to completing the cross-country phase.
2. National Dressage Championships, Ireland.
  Data were collected from horses and ponies (n = 143) competing at the Dressage Ireland National Dressage Championships run over three days. Permission was sought from riders as they left the arena after completing their dressage test.
3. International CCI* and CCI** event, UK.
  This FEI event was conducted under FEI and British Eventing regulations and run over three days. All horses were subjected to a tack inspection, carried out by FEI tack stewards immediately following the dressage phase of the event. Data were collected from horses and ponies (n = 213) immediately following the official tack inspection during two days of the competition.
4. National Dressage Championships, Belgium
  This event was run and organised by the FEI, under FEI regulations over three days. All horses were subjected to a tack inspection carried out by FEI tack stewards immediately following the dressage test. Data were collected from horses and ponies (n = 126) immediately following the official tack inspection during two days of the competition.
5. Performance Hunter Classes, Ireland
  Data were collected during performance hunter classes for both Connemara ponies and Irish Draft horses (n = 62). This competition was organized and regulated by the FEHL. Data were collected during a tack inspection carried out immediately prior to competing during one day.
6. International Dressage CDI 3* show, Belgium
  This event was conducted under FEI regulations and run over a six day period. All horses were subjected to a tack inspection, carried out by FEI tack stewards immediately following the dressage test. Data were collected from horses (n = 54) immediately before the official tack inspection during one day of the competition.

All noseband measurements and tightness level assessments were carried out by the first author (OD). An assistant recorded data including noseband type during competitions listed 1–3 above. All data were recorded by the first author during competitions 4–6. The competition venues were chosen on the basis of permission being given by the organizers, officiating FEI stewards or governing bodies. A written outline of the purpose of the data collection was placed immediately adjacent to the location of data collection at each location. Verbal queries were addressed as they arose. Riders were not obliged to permit data collection if they did not wish to participate in the study. A small number of riders (n = 13) were unwilling to participate in this study. A check-sheet and clip-board were used on each occasion to record data on noseband tightness, width, position and noseband type. Data were collected between June 2013 and March 2016 and stored in a spreadsheet (Microsoft Excel, 2010).

**Fig 2:**
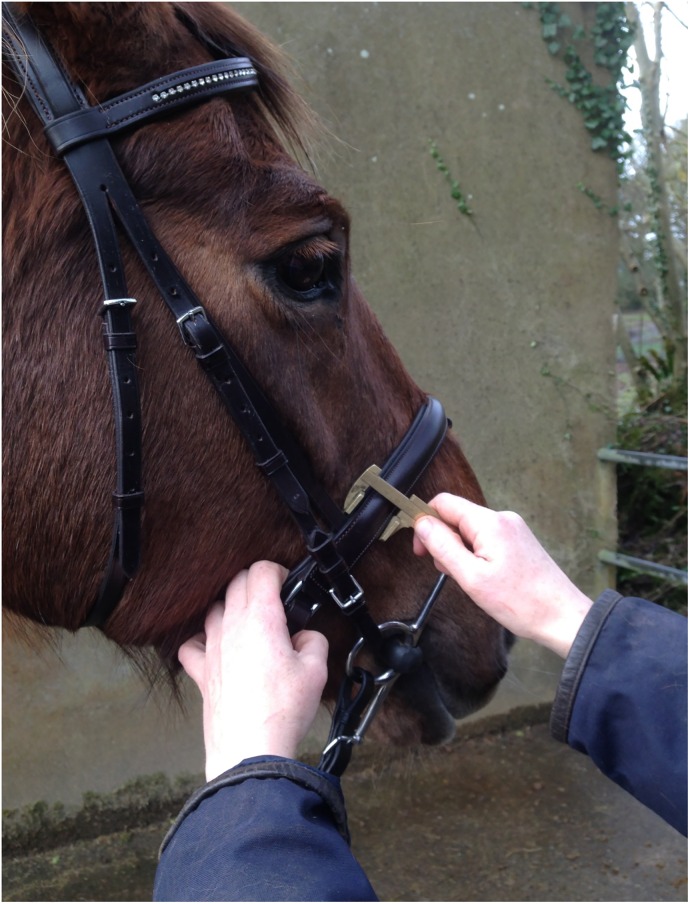
Use of TB callipers to measure the width of the noseband (mm).

**Fig 3:**
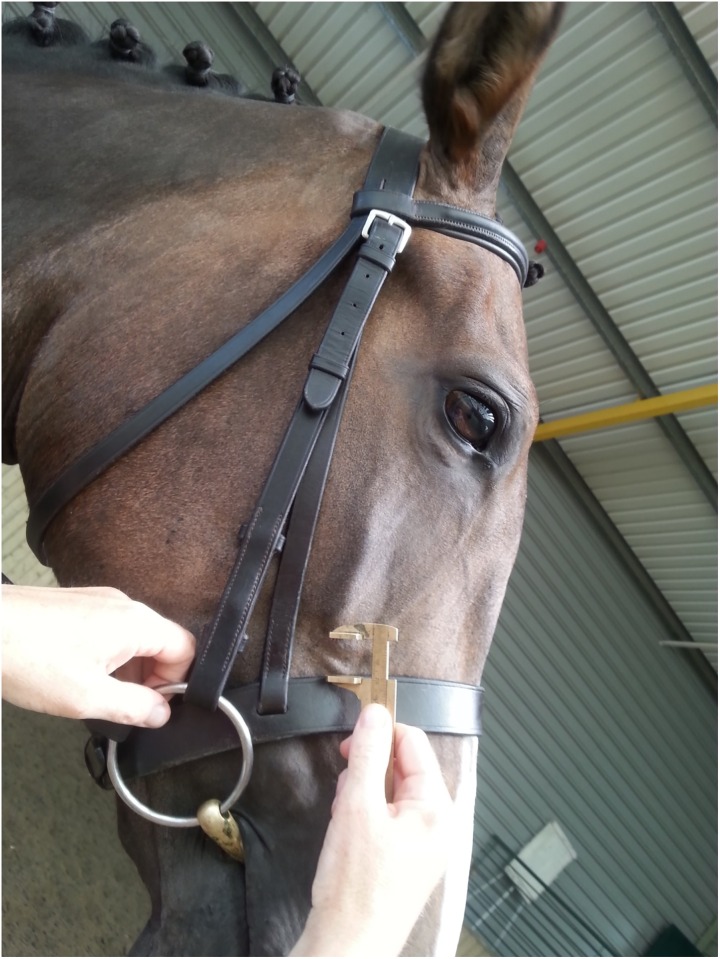
Use of TB callipers to measure distance (mm) between rostral margin of facial crest and caudal margin of noseband.

The following data were collected and recorded:

1. Date and competition (or class in which the horse was competing).
2. Age of horse–where available. Recording of horse age was not possible for all classes but age data were recorded for the young event horse classes and the international event.
3. Horse and rider details were recorded to identify horses that had been presented at previous competitions. Repeat measurements were not taken on individual horses.
4. Type of noseband. This study did not differentiate between different types of cavesson noseband and therefore crank nosebands were assigned to the same group as the standard cavesson.
5. Width of the noseband in mm: this was measured by placing the arms of the callipers on the caudal and rostral margins of the noseband lateral to the nasal bone, approximately level with the ventral edge of the facial crest (Fig 2).
6. Distance between the ventral edge of the facial crest on the right hand side of the face and the upper (caudal) margin of the noseband immediately distal to the facial crest was measured using the callipers (Fig 3). The measurement was not recorded for either grackle nosebands (n = 49) or Micklem bridles (n = 26) as the position of the noseband in both of these was above the ventral edge of the facial crest.
7. Tightness of the noseband. The tightness of the noseband was assessed in the midline of the nasal planum, by sliding the ISES Taper Gauge under the noseband, in a rostro-caudal direction as far as it would progress without causing dorsal displacement of the noseband on the nose, or elevation of the horse’s head. Dorsal displacement of the noseband on the nose or elevation of the horse’s head were regarded as evidence of excessive force.

Noseband tightness was categorised as follows:

- Zero fingers (0 F): the noseband was too tight to allow the tip of the taper gauge to be inserted beneath the noseband.
- 0.5 fingers (0.5 F) = the tip of the taper gauge could be introduced beneath the noseband but the gauge could not be moved upward under the noseband to the 1 finger notch without excessive force.
- 1 finger (1.0 F) the taper gauge could be moved comfortably to the 1 finger notch, but no further without excessive force.
- 1.5 fingers (1.5 F) the taper gauge could be moved easily beyond the 1 finger notch but not as far as the 2 finger ridge without excessive force.
- 2.0 fingers (2.0 F the taper gauge could be easily moved to the 2 finger ridge with minimal force.
- Nosebands fastened so loosely as to permit the taper gauge to move easily beyond the 2 finger ridge were recorded as greater than 2.0 fingers.

## Data Analysis

Analysis was carried out using SPSS (IBM SPSS Statistics, Version 22, 2015). Kolmogorov-Smirnov values were generated to test the data for normality. The Kruskall-Wallis test was used to compare noseband tensions in each of the three disciplines of eventing, dressage and performance hunter. Mann-Whitney tests were used to compare noseband tightness levels between disciplines, competition type and age of horse. Mean ± standard error of the mean, and also median and interquartile ranges for noseband width and distance between rostral margin of the facial crest and dorsal margin of the noseband were calculated.

## Results

Data were collected from 750 horses and ponies (S1 Dataset), of which 47% (n = 354) were competing in eventing, 45% (n = 334) were competing in dressage and 8% (n = 62) were competing in performance hunter classes. Noseband width values had a non-parametric distribution (D(663) = 0.07, p<0.001). Measurements of distance between the facial crest and the proximal (upper) margin of the noseband were not normally distributed (D(619) = 0.08, p<0.001. Noseband tensions were not normally distributed (D(737) = 0.281, p<0.001).

### Noseband type

**Fig 4:**
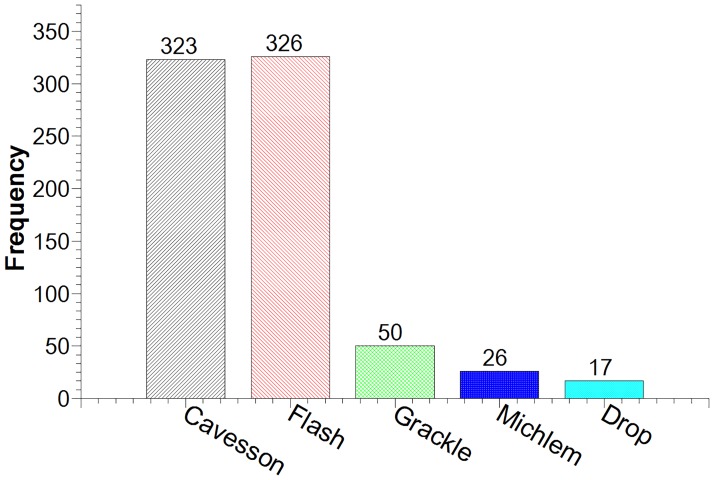
Frequency of usage of five different noseband types.

The flash was the most common noseband (43.4%, n = 326) and the drop noseband the least common (2.3%, n = 17) (Fig 4).

### Noseband width

**Fig 5:**
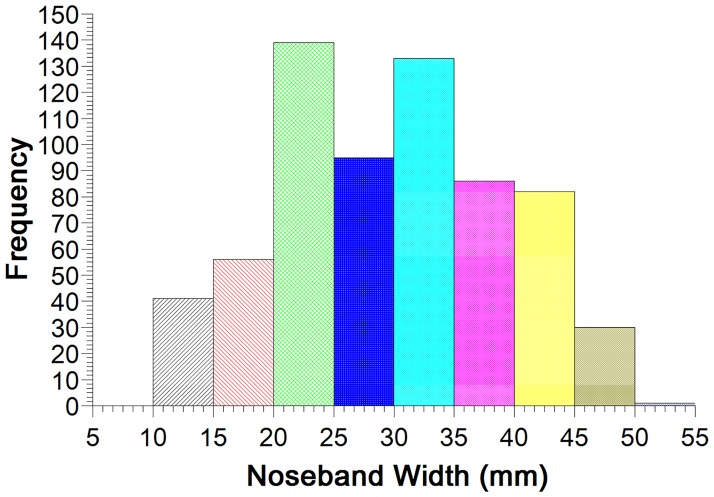
Distribution of noseband widths.

**Fig 6:**
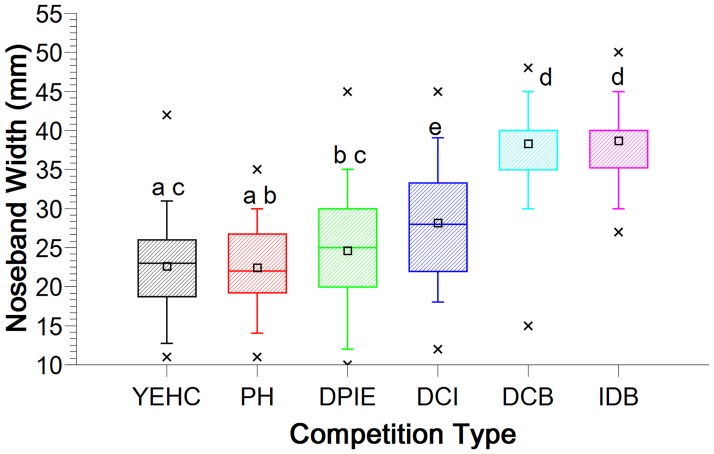
Boxplot of noseband width and interquartile range for six competition types. Widths differ significantly except where they share a superscript. Maximum and minimum values are indicated by ‘x’, with whiskers identifying the 5% and 95% values. The large rectangles represent 25–75% of values, with the median value indicated by the thick crossline, and mean values by the small square symbol.

Noseband width ranged from 10–50 mm (median = 30 mm, IQR = 22, 35) (Fig 5). Noseband width was greatest in horses in dressage competitions (Fig 6).

### Noseband position

**Fig 7:**
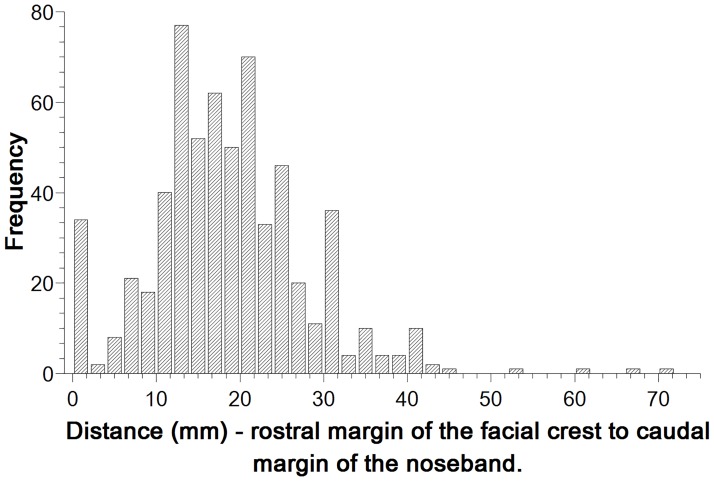
Frequency distribution of distances (mm) between the rostral margin of the facial crest and the caudal margin of the noseband (n = 619).

**Fig 8:**
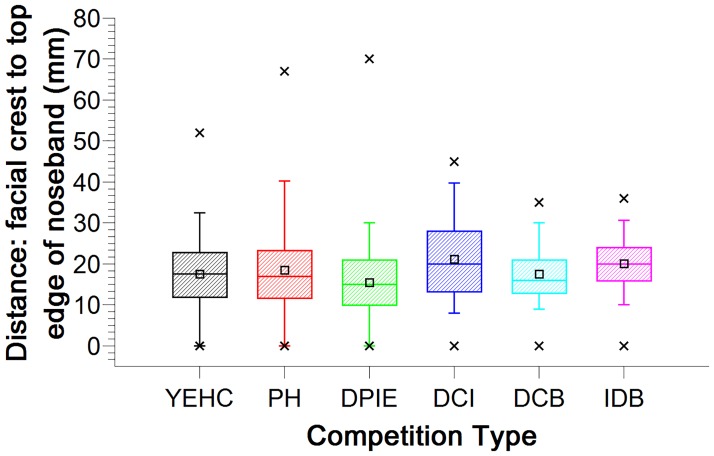
Boxplot and interquartile range of distance from rostral margin of facial crest to dorsal margin of noseband (mm) for six competition types. Maximum and minimum values are indicated by ‘x’, with whiskers identifying the 5% and 95% values. The large rectangles represent 25–75% of values, with the median value indicated by the thick crossline, and mean values by the small square symbol.

The median noseband position was 17mm from the facial crest and measurements ranged from 0 mm to 70 mm (Fig 7). There was no significant difference in this measurement between noseband types (H = 3.65, p = 0.45). There was a significant difference in the distance of the noseband to the facial crest within competition type (Fig 8). The noseband–to-crest distance was significantly less for the dressage phase of the international event than it was for both the dressage championships, Ireland (U = -107.03, p < 0.001) and the international (CDI) dressage competition in Belgium (U = -109.04, p < 0.005). No other significant differences were found between competition types.

Dressage championships, Ireland had the highest mean noseband-to-crest distance (21 ± 1 mm) with a median of 20 mm and an interquartile range of 13–28 mm, followed by the international (CDI) dressage competitions, Belgium (20 ± 1 mm) with a median of 20 mm and an interquartile range of 16–24 mm. Young Event Horse classes had a mean noseband-to-crest distance of 18 ± 1 mm, with a median of 18 mm and an interquartile range of 12–23 mm, while Performance Hunter lasses had a mean noseband-to-crest distance of 19 ± 2 mm with a median of 17 mm and an interquartile range of 11–23 mm. The lowest mean noseband-to-crest distances were in the dressage phase of the international event (16 ± 1 mm) with a median of 15 mm, an interquartile range 10–21 mm and in the dressage championships in Belgium with a mean of 18 ± 1 mm, a median of 16 mm and an interquartile range of 13–21 mm (Fig 8).

### Noseband tightness

**Fig 9:**
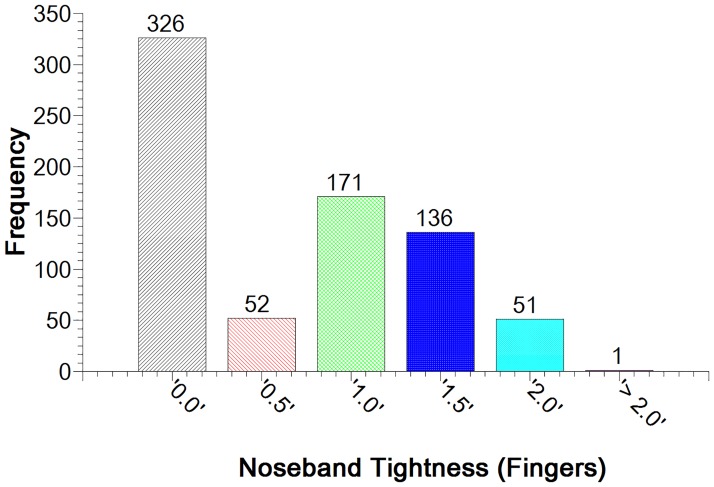
Distribution of noseband tightness measurements found in horses (n = 750) competing in the disciplines of dressage, eventing and performance hunter.

The median noseband tightness in all horses measured (n = 737) was found to be 0.5 fingers. Forty four per cent of nosebands were tightened to zero fingers tightness, 7% to 0.5 fingers, 23% to 1 finger, 19% to 1.5 fingers and 7% to 2 fingers tightness (Fig 9).

Noseband tightness measurements for individual disciplines were not normally distributed (eventing: D(352) = 0.328, p<0.001, dressage: D(323) = 0.241, p<0.001, performance hunter: D(62) = 0.224. p<0.001). There were significant differences in noseband tightness levels among the three disciplines (eventing, dressage and performance hunter) (H(2) = 27.99, p<0.001). Tightness levels of nosebands were highest in eventing competitions (Mdn = 0.0) (n = 352), then in dressage competitions (n = 323) (Mdn = 1.0, p< 0.001) and were lowest in performance hunter classes (n = 62) (Mdn = 1.0, p<0.001).

**Fig 10:**
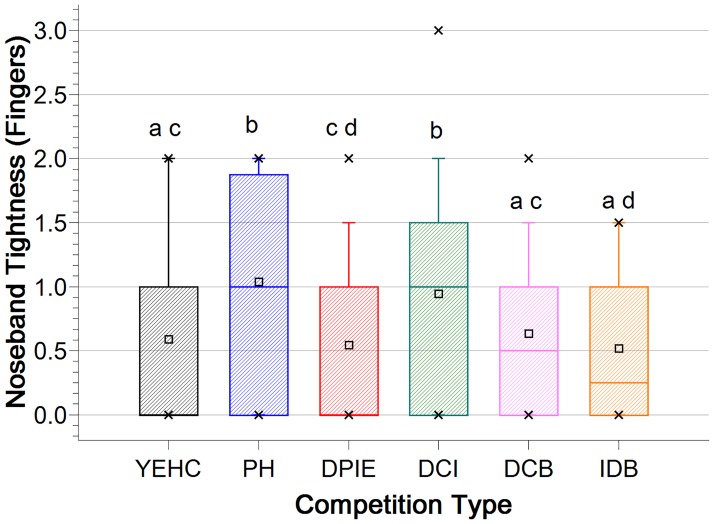
Median and inter-quartile range measures of noseband tightness level (Fingers) in 6 different competition types. Tightness levels differ significantly except where they share the same superscript. Maximum and minimum values are indicated by ‘x’, with whiskers identifying the 5% and 95% values. The large rectangles represent 25–75% of values, with the median value indicated by the thick crossline, and mean values by the small square symbol.

There were significant differences in noseband tightness levels between different competition types (H = 47.34, df = 5, p<0.001) (Fig 10).

Adjusted significance p values for multiple comparisons of noseband tension between competition types are shown in Table 1.

**Table 1:** Pairwise comparisons of noseband tension levels between different competitions.

Competition	Mann- Whitney U	Adjusted Significance P value
**Dressage phase of international event—Dressage Championships Ireland**	-104.39	0.001
**Dressage phase of international event—Performance Hunter**	124.18	0.001
**Young event horse class—Dressage Championships Ireland**	-95.04	0.001
**Young event horse class—Performance Hunter**	-114.83	0.01
**Dressage Championships Belgium—Performance Hunter**	98.95	0.006
**Dressage Championships Belgium—Dressage Championships Ireland**	79.17	0.005
**International Dressage (CDI) Belgium- Dressage Championships Ireland**	119.59	0.003
**International Dressage (CDI) Belgium- Performance Hunter**	140.34	0.003

Within eventing competitions, no significant difference was found in noseband tightness level between horses competing in the dressage phase (n = 213) and at the cross-country phase (n = 139) (U = 9.35, p>0.05). The horses sampled and competing in the cross-country phase of the event were all competing in a league run for young event horses only, so data were collected from either four or five year old horses. These had noseband tightness levels which were not significantly different to those at the dressage phase (age range 6–19 years). Similarly, comparison of noseband tightness between four year old horses (n = 80) and five year old horses (n = 59) found non-significant differences although the tightness levels in the older horses were higher (U = 2064, p>0.05).

**Fig 11:**
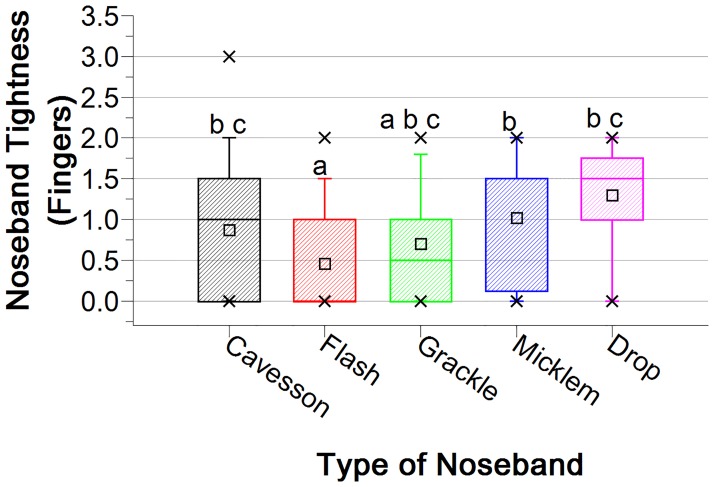
Noseband tightness measured in five different noseband types. Tightness levels differ significantly except where they share the same superscript. Maximum and minimum values are indicated by ‘x’, with whiskers identifying the 5% and 95% values. The large rectangles represent 25–75% of values, with the median value indicated by the thick crossline, and mean values by the small square symbol.

Significant differences in noseband tightness were found between the five different types of noseband used by competitors (H = 74.89, p<0.001). The flash noseband was found to be significantly tighter than the drop noseband (U = -239.28, p<0.001) and also the cavesson (U = 117.99, p<0.001) and Micklem (U = -164.23, p<0.005) (Fig 11).

Differentiation was not made between different cavesson noseband types. Therefore, the cavesson results included both plain and crank cavesson nosebands. Median, interquartile range, mean and standard error values of noseband tightness for five different noseband types are shown in Table 2.

**Table 2:** Median, interquartile range, mean and standard error values of noseband tightness levels found for five different noseband types.

Noseband Type	Median	Interquartile range	Mean	SE
**Cavesson**	1.0	(0.0, 1.5)	0.9	0.04
**Flash**	0.0	(0.0, 1.0)	0.5	0.03
**Grackle**	0.5	(0.0, 1.0)	0.7	0.09
**Micklem**	1.5	0.0, 1.5)	1.0	0.14
**Drop**	1.5	(1.0, 2.0)	1.3	0.17

## Discussion

This study shows that 44% of competition horses in dressage and eventing competitions at national and international level, under the regulations of the FEI, British Eventing, Dressage Ireland and the Future Event Horse League, had nosebands tightened to such an extent that it was not possible to insert the ISES taper gauge under the noseband (classified as zero fingers’ tightness). This indicates a widespread tendency to tighten the noseband to a substantially higher level of tightness than that suggested in equestrian texts [4–9]. Over half of all nosebands tested were tightened to 0.5 fingers or tighter. Only 7% of nosebands were fitted at the tightness level of 2.0 fingers, and only one noseband (0.1%) was at greater than 2.0 fingers tightness level. The methodology did not allow for more exact measurements than those possible using the ISES taper gauge but the use of the taper gauge does ensure that the results of the current study are relevant to those considering the ISES position statement and historic advice. The findings, grouped into six tightness levels, do give an indication of current noseband usage trends and highlight the need for further research into riders’ motivations to tighten nosebands excessively.

Regulations within some equestrian disciplines prohibit certain noseband types while others, such as elite dressage competitions mandate double bridles with cavesson noseband (of which the crank type is the most common). Crank nosebands allow a doubling of the tightness achievable for a given amount of handler tightening effort.

The tightest nosebands were found among eventing horses. This is not entirely surprising since the control of a horse is inherently more challenging as the horse is ridden at speed towards, and over, obstacles and over uneven terrain [18]. However, any use of relentless pressure defies the principles of learning theory since it does not provide an opportunity to release the pressure and condition the horse through negative reinforcement [15]. Thus, riders who come to rely on tight nosebands are effectively training their horses to work only with such devices.

For performance hunter classes, horses are ridden over a variety of obstacles that can include show-jumping and cross-country type fences. Competitors are also required to execute basic ridden movements similar to a simple dressage routine which are often performed immediately after completion of the show-jumping phase. Horses are frequently ridden in a show-type bridle which typically includes a plain cavesson noseband, without the crank functionality. The absence of the crank function may, in part, explain the lower noseband tightness found in the performance hunter class in this study.

This study reveals the prevalence of restrictive noseband usage on competition horses of all ages. Noseband tightness levels do not appear to be influenced by the stage of training or the particular traits of the horse being ridden since tightness did not differ significantly between young and older event horses in the study. The widespread use of tightness levels of less than two fingers may be indicative of habitual or routine over-tightening of nosebands as a pre-emptive response rather than as a consequence of previous training or control problems.

The findings of this study indicate that most competition riders, in the disciplines of dressage and eventing, in at least three European countries use tight nosebands. However, some equestrian sports such as reining show no reliance on nosebands. In the absence of regulations or guidelines outlining recommended or permitted noseband tension in competition, competitors are free to adjust the noseband to the tightness level that they deem necessary or appropriate. The widespread use of tight, i.e. less than 2.0 fingers, nosebands in three European countries and across the disciplines of eventing and dressage under FEI and national federation regulations points to the need for similar, more extensive studies to establish the prevalence of this practice worldwide. The lack of regulation of noseband tightness in competition may reflect the lack of available data on noseband usage and on the possible consequences of excessively tight nosebands.

Several reasons for using a tight noseband have been suggested. A tight noseband may reduce the likelihood of horses opening their mouths [12]. Mouth-opening is penalised if it occurs during dressage competition, as it is interpreted as a resistance or evasion [16, 19]. Oral movements, such as putting the tongue over the bit or moving the position of the bit in the oral cavity, are likely to be inhibited by restrictive nosebands [8, 20, 1]. Such movements, often called evasions, may reduce the impact of bit pressure and thus compromise rider control. Preliminary research on the relationship between noseband tightness and response to bit pressure indicates that tight nosebands result in increased sensitivity to bit pressure [13, 21]. This effect may be as a consequence of compression of the oral structures including the lips and tongue. In a UK survey, 84% of riders (n = 790) reported lack of responsiveness to bit pressure, including not slowing down when asked to, and jogging when asked to walk [22]. The current findings may reflect riders’ efforts to address such problems, at least partly, by tightening nosebands, rather than putting the required time into training horses to slow, stop or stand still from a rein cue [23].

Mouth-opening is thought to be a manifestation of oral discomfort [24] and can be a response to increased rein tension [25]. Possible adverse consequences of excessive tightening of the noseband include restriction of mouth-opening and other normal behaviours [2], pain, tissue damage, buccal ulceration/laceration as a consequence of buccal mucosa being pressed against the sharp edges of premolar teeth [26] and some restriction on breathing [8].

Few data are yet available on the physiological effects of noseband pressure on the sub-noseband tissues of horses. Some information on the effects of compressive devices such as tourniquets and ligatures on human tissue is available [27–28] which may provide some guidance for future equine studies. Improper tourniquet use can result, for instance, in neuron damage after compression at 50 mmHg over two hours, with more damage at higher pressures [29]. Following the use of a tourniquet, electromyographic and functional evidence of damage to nerve conduction and muscle function has been reported in 71% of patients [30]. Similarly, some degree of impaired sciatic function in dogs is associated with every tourniquet application [31]. Pain caused by pressures of 300–400 mmHg resulted in half of the human participants being withdrawn from a tourniquet study [32]. A tight noseband exerts pressure directly on superficial anatomical structures of the horse’s head. These include several muscle groups, the lateral nasal artery and vein and the facial and infraorbital nerves. If the pressures exerted by a noseband result in neural damage, possible cumulative effects include denervation (and subsequent desensitization of an area) or trigeminal neuritis, a condition thought to be associated with head-shaking in horses [33–35]. While desensitisation through anaesthesia of local nerve supply is useful in medical practice, the consequence in equitation may be a reduced sensitivity to facial and oral pressures exerted by riders.

## Conclusion

Tight nosebands are a common feature of equestrian competition, with almost half of eventing and dressage competitors assessed in Ireland, England and Belgium tightening the noseband to zero fingers tightness level. Only seven per cent of nosebands assessed allowed two fingers to be placed beneath the noseband at the nasal planum, with the remainder of nosebands being fitted tighter than this. This practice was not influenced by the age of the horse. The current study highlights the need for quantification of pressures exerted by tight nosebands.

## Supporting Information

S1 Dataset Noseband_Data.xlxs. Noseband type, width, position and tightness recorded on 750 horses in national and international level competition in eventing, dressage and performance hunter classes in Ireland, England and Belgium.
(XLSX)
